# Predicting 72-Hour Fatality in Severe Hyperphosphatemia: A Comparative Analysis of Multivariate Logistic Regression and Machine Learning Models in a Single-Center Study

**DOI:** 10.7759/cureus.80734

**Published:** 2025-03-17

**Authors:** Keishiro Sueda, Susumu Ookawara, Kai Saito, Takahiko Fukuchi, Kiyoka Omoto, Hitoshi Sugawara

**Affiliations:** 1 Comprehensive Medicine, Saitama Medical Center, Jichi Medical University, Saitama, JPN; 2 Laboratory Medicine, Saitama Medical Center, Jichi Medical University, Saitama, JPN

**Keywords:** calibration, critical value, evaluation study, fatal mortality, hyperphosphatemia, logistic model, machine learning, outlier value, roc curve

## Abstract

Background: Hyperphosphatemia is associated with several serious diseases, including chronic kidney disease, tumor lysis syndrome (TLS), rhabdomyolysis, sepsis, and acute respiratory distress syndrome. This study investigates the critical issue of predicting 72-hour fatality in patients with severe hyperphosphatemia (≥ 10 mg/dL).

Methods: We analyzed data from 530 patients treated at the Saitama Medical Center, Japan, from 2004 to 2019, including 153 72-hour fatalities. Multivariate logistic regression analysis (MLRA), Prediction One™ (Sony Network Communications Inc., Tokyo, Japan, https://predictionone.sony.biz/), and Light Gradient Boosting Machine (LightGBM) were used to predict fatalities. These methods were evaluated on a validation set of 331 patients from 2020 to 2023, including 104 fatalities. Calibration plots for training and validation data were used for comparison.

Results: The fatality rate was 28.9% in the training data and 31.4% in the validation data. MLRA identified five fatality factors: age, low albumin, high aspartate aminotransferase, and elevated potassium and magnesium levels, with an area under the curve (AUC) of 0.848 (95% CI: 0.801, 0.890), sensitivity of 0.862, and specificity of 0.704. Prediction One™ achieved an AUC of 0.770 (95% CI: 0.722, 0.818), sensitivity of 0.654, and specificity of 0.769. LightGBM achieved an AUC of 0.948 (95% CI: 0.923, 0.973), sensitivity of 0.863, and specificity of 0.889. The validation calibration plot showed that MLRA had the closest regression coefficient to 1.0 at 0.903.

Conclusion: Although MLRA was the most accurate in predicting 72-hour fatalities, machine learning methods provided valuable insights into the importance of variables. Considering the high mortality rates associated with severe hyperphosphatemia, timely and accurate prognostication is essential in guiding immediate interventions and improving outcomes in emergency settings.

## Introduction

Healthcare facilities generate laboratory data daily, which can be used to improve healthcare management. However, the comprehensive use of laboratory databases to enhance medical care quality remains suboptimal. Critical values represent pathophysiological states that deviate significantly from the normal range, necessitating immediate action to prevent life-threatening conditions [1]. Extreme outlier values in laboratory tests are defined as those falling below the 0.5-1.0 percentile or exceeding the 99.0-99.5 percentile, in accordance with the Japanese Society of Laboratory Medicine Guideline [2]. Several studies have demonstrated an association between outliers and short-term mortality [3-5].

Phosphate homeostasis is regulated through a complex interplay of intestinal absorption, renal tubular reabsorption, and phosphate exchange between cells and bone stores [6]. Hyperphosphatemia, a common biochemical abnormality affecting approximately 30% of hospitalized patients [7], is frequently observed in chronic kidney disease [8], tumor lysis syndrome (TLS) [9], rhabdomyolysis [10], sepsis [11,12], and acute respiratory distress syndrome [13]. It contributes to increased mortality through calcium-phosphate precipitation, vascular calcification, myocardial dysfunction, and systemic inflammation [14]. Additionally, studies have linked hyperphosphatemia to worse outcomes in pneumonia [15] and cardiovascular diseases [16]. Given these risks, early identification and timely intervention are critical to preventing complications and improving patient outcomes.

Severe hyperphosphatemia (≥10 mg/dL; 3.23 mmol/L) is a recognized indication for hemodialysis in TLS, particularly in cases complicated by refractory electrolyte disturbances (e.g., severe hyperkalemia, K>6.0 mEq/L, or symptomatic hypocalcemia) or worsening renal function (serum creatinine (Cr) >10 mg/dL or serum uric acid (UA) >10 mg/dL) [17]. In non-TLS patients, the optimal timing for dialysis initiation remains unclear [18]. Clinical decisions regarding hemodialysis are typically based on the presence of severe hyperkalemia, congestive heart failure, or symptomatic uremia due to advanced renal dysfunction.

However, evidence to accurately predict the clinical factors related to early fatality among patients with severe hyperphosphatemia (≥ 10 mg/dL) is limited. Only a few studies have identified factors associated with predicting 72-hour mortality with severe hyperphosphatemia [19]. Hence, additional evidence is required to inform appropriate early clinical management decisions for interventions in treating elevated serum phosphate in primary and critical care settings. Therefore, this study investigated the correlation between 72-hour fatality after the test date and predictive factors among patients diagnosed with severe hyperphosphatemia.

We employed multivariate logistic regression analysis (MLRA), Light Gradient Boosting Machine (LightGBM), and Prediction One™ (Sony Network Communications Inc., Tokyo, Japan, https://predictionone.sony.biz/) to develop and compare predictive models for binary classification in a dataset with missing values. These models were selected based on their ability to handle missing data while ensuring predictive accuracy, computational efficiency, and interpretability. MLRA was chosen for its interpretability, providing odds ratios (OR) and statistical significance for each predictor. It was also used to identify the characteristics of these patients, offering insights into the key clinical factors associated with the outcome. Since MLRA does not natively support missing data, listwise deletion was applied to include only complete cases [20]. In contrast, LightGBM [21] was selected for its native handling of missing values and categorical variables, eliminating the need for explicit imputation or encoding. Unlike random forest, which requires categorical variables to be numerically transformed before use, LightGBM processes them directly within its decision tree learning framework. Additionally, Prediction One™ was included as an automated machine-learning platform that natively handles missing data and categorical variables. It generates predictive models without requiring programming or extensive preprocessing, making it a practical tool for clinical research [22].

Understanding the factors associated with initial 72-hour fatality in patients with severe hyperphosphatemia is essential to guide clinical decisions in critical care. We hypothesized that a novel 72-hour prognosis prediction model for patients with high serum phosphate levels would assist physicians in decision-making. Additionally, we determined which method of model construction yielded high accuracy. Our findings could offer therapeutic options and improve initial medical management.

## Materials and methods

Study design

**Figure 1:**
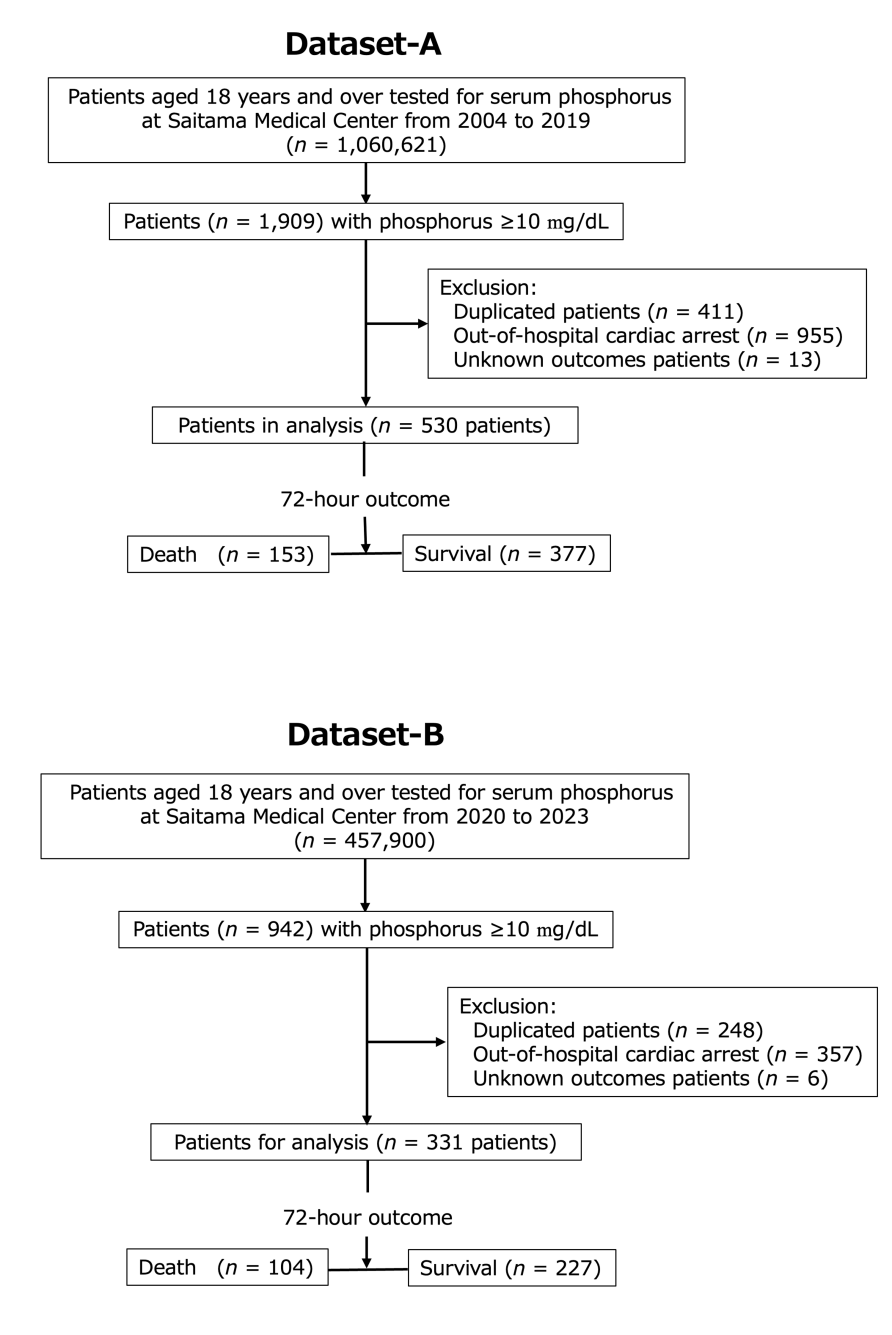
Flow diagram outlining patient selection process. We identified a cohort of 1,060,621 patients who underwent serum IP testing at the clinical laboratory of our medical center over 16 years, from 2004 to 2019. Of these, 1,909 patients had severe hyperphosphatemia (≥10 mg/dL). We excluded 411 metachronous duplicates (retaining only the highest serum IP value for each patient), 955 patients with out-of-hospital cardiac arrest, and 13 with unknown outcomes. This resulted in a final sample of 530 patients who were enrolled in this study (Dataset-A) for the derivation dataset. For the validation dataset, we compiled Dataset-B, composed of 331 patients with severe hyperphosphatemia (≥10 mg/dL) from 2020 to 2023, including 104 deaths and 227 survivals, selected using the same criteria as for Dataset-A. IP, inorganic phosphorus

This study was a single-center retrospective inception cohort study. We defined severe hyperphosphatemia as ≥10 mg/dL, a criterion for initiating hemodialysis in TLS [17], and the exclusion criteria in randomized controlled trials of hemodialysis in hyperphosphatemia [23]. We used the descending order of serum inorganic phosphorus (IP) data list, displaying all 1,060,621 patients from the clinical laboratory of our medical center from 2004 to 2019. We extracted data from 1,909 patients over 18 years of age with severe hyperphosphatemia, with a 0.18% occurrence rate. In the case of multiple entries, only the highest IP from each patient was included. In addition, patients with out-of-hospital cardiac arrest and unknown outcomes due to transfer to another hospital were excluded from the chart review. A sample of 530 patients was selected as the derivation dataset (Dataset-A) to construct a model to predict 72-hour fatality after applying the exclusion criteria. Dataset-B, comprising 331 patients with severe hyperphosphatemia from 2020 to 2023, selected as in Dataset-A, was used to validate the model externally. Figure 1 illustrates the flowchart of the chosen cohorts. The detailed study protocol is provided in the Appendices.

Sample size estimation

The following parameters were applied to compute the required sample size using G*Power [24]: test family: z tests; statistical test: logistic regression; type of power analysis: a priori: compute required sampling size, given α, power, and effect size; tail, two; P(Y=1 |X=1)H1=0.28 (i.e., an assumption based on the probability of death (Y=1) for patients whose serum phosphorus (≥10 mg/dL) (X=1) was P1=0.28); P(Y=1|X=1) Ho=0.02 (i.e., an assumption based on the probability of death (Y=1) for patients with serum phosphorus under 10 mg/dL (X=1) was P0=0.01); α error=0.05; power=0.8; R-squared other X=0.09 (i.e., death assumption based on moderate association with serum phosphorus level (say R=0.30); X-distribution: binominal; X param ∏:0.02 (i.e., proportion of cases with serum phosphorus ≥10 mg/dL). These fraternity assumptions were based on a previous study [25]. The sample size was calculated as 517. We expanded the data size to 530 to achieve high power.

Primary outcome and endpoints

The primary outcome was defined as death within 72 hours of IP testing, whether the patient was hospitalized or ambulatory. Cases were defined as patients with IP≥10 mg/dL who died in the hospital during the first 72 hours after the test. Controls were defined as patients with IP≥10 mg/dL who survived. The index date of the inception cohort was defined as the point in time with the highest IP.

Issue of interest

The association of the following characteristics with the 72-hour fatality outcome was analyzed: age, sex, height, weight, body mass index, number of cigarettes smoked (Brinkman index); vital signs at examination time, including systolic blood pressure (SBP), diastolic blood pressure (DBP), pulse rate (PR), respiratory rate (RR), and body temperature (BT); laboratory test values, including white blood cell count (WBC), hemoglobin (Hb), hematocrit (Ht), platelet count (Plt), total protein (TP), albumin (ALB), total bilirubin (T-Bili), direct bilirubin (D-Bili), aspartate transaminase (AST), alanine transaminase (ALT), γ-glutamyl transpeptidase (γ-GTP), lactate dehydrogenase (LD), alkaline phosphatase (ALP), creatine kinase (CK), amylase, C-reactive protein (CRP), serum IP, sodium, potassium, chloride, calcium, magnesium, blood urea nitrogen (BUN), Cr, UA, total cholesterol (TC), triglyceride (TG), high-density lipoprotein cholesterol (HDL-C), random plasma glucose (RPG), prothrombin time-international normalized ratio (PT-INR), activated partial thromboplastin time (APTT), antithrombin III, fibrinogen, fibrin/fibrinogen degradation products (FDP), and D-dimer; updated Charlson comorbidity index (CCI) total score [26], CCI components, expected causes of extremely high IP, and presence or absence of dialysis before or after severe hyperphosphatemia. Dialysis considered included intermittent hemodialysis, peritoneal dialysis, and continuous renal replacement therapy.

Routine laboratory tests

Routine hematological tests were performed on an automated hematology system (XN-3100TM; Sysmex Corporation, Kobe, Japan) at the Department of Laboratory Medicine, Jichi Medical University, Saitama Medical Center. Routine biochemical tests were performed on an automated biochemical analyzer (BioMajesty™ series JCA-BM6010/C; JEOL Ltd., Akishima, Japan), and coagulation tests were performed using an automated coagulation analyzer (CP3000TM; Sekisui Medical Co., Ltd., Tokyo, Japan), HbA1c levels were measured using the BioMajesty™ series JCA-BM6010 G type (BioMajesty™ series JCA-BM6010/C; JEOL Ltd., Akishima, Japan).

Internal and external quality control (IQC) measures are rigorously implemented to ensure the accuracy and reliability of clinical laboratory testing. For IQC, the same sample is measured multiple times within a single day to assess intra-day variability, and across consecutive days to evaluate inter-day variation. A certified clinical laboratory physician, accredited by the Japanese Society of Laboratory Medicine, reviews these data to ensure analytical precision. For external quality control (EQC), the laboratory participates in nationally and regionally recognized proficiency testing programs, including those conducted by the Japanese Association of Medical Technologists, the Japan Medical Association, and the Saitama Medical Association. These programs provide objective assessments of analytical performance and ensure standardization across institutions. The hospital’s quality control framework, integrating IQC and EQC, is accredited by the Japan Council for Quality Health Care (JCQHC; Accreditation Number: JC932-4), confirming compliance with national standards and a commitment to high-precision clinical testing.

Ethical approval

The study protocol was designed according to the tenets of the Declaration of Helsinki [27]. The Institutional Clinical Research Ethics Review Board of Saitama Medical Center, Jichi Medical University, Saitama, Japan, approved this study (Clinical #S10-79, #S20-025, and #S21-147) and waived the requirement to obtain informed consent because of the retrospective study design.

Statistical analysis

Descriptive Statistics

We created cross tables dividing the outcomes into survivals and deaths. Summarized values of all variables are presented as median and interquartile range. Differences between the outcomes in the derivation dataset were tested using Fisher’s exact test or Pearson’s chi-square test for nominal variables and the Mann-Whitney U-test for continuous variables.

A Chi-square test for homogeneity was performed to compare the proportion of patients receiving dialysis, defined as intermittent hemodialysis, peritoneal dialysis, and continuous renal replacement therapy, between Dataset-A and Dataset-B, stratified by the timing of dialysis initiation (before or after the onset of severe hyperphosphatemia). Additionally, a Chi-square test for independence was conducted to assess whether the proportion of patients undergoing dialysis before or after the onset of severe hyperphosphatemia differed significantly between deceased and surviving patients within each dataset.

Additionally, we examined the 72-hour fatality rate by determining the serum phosphorus concentration for derivation and validation data.

Association Analysis Using Logistic Regression Analysis

A normal probabilities plot was employed to determine the normality of all continuous laboratory test values and assess the residual error of all continuous variables. Continuous variables deviating from the normal distribution were transformed using the Box-Cox formula before logistic regression analysis [28]

$X=\frac{x^{\lambda}-1}{\lambda}...(\lambda\neq 0.0); X=log(x)...(\lambda=0.0),$

where x and X are the test results before and after transformation, respectively, and λ is the transformation parameter.

The power used for laboratory tests was λ=0.0 (log-transformation) for Hb, TP, T-Bili, D-Bili, AST, ALT, γ-GTP, LD, ALP, CK, amylase, CRP, IP, magnesium, UA, TC, TG, HDL-C, RPG, PT-INR, APTT, fibrinogen, antithrombin III, FDP, and D-dimer. It was λ=0.5 for WBC.

Univariate logistic regression analysis was used to determine the potential factors related to 72-hour fatality and calculate odds ratios (OR) and 95% CIs. The area under the curve (AUC) of significant covariates was calculated using receiver-operating characteristic (ROC) analysis. MLRA was performed for covariates significantly in univariate analysis, estimating the OR with a 95% CI adjusted for confounders such as age and IP level.

Model Development of Statistical and Two Machine Learning Methods

Model development followed the transparent reporting of a multivariate predictive model for individual prognosis or diagnosis plus artificial intelligence statement [29]. An MLRA model for predicting 72-hour fatality (Model 1) was developed using a variable reduction method, starting from a full model including all candidate continuous variables. Listwise deletion was used in cases of missing values. For actual variable selection, efforts involved maximizing the number of valid data points, ensuring at least 10 events (death) per variable, increasing the AUC, and decreasing the Akaike information criterion (AIC) [30]. A variance inflation factor (VIF) of five or more indicated multicollinearity, excluding one of the correlated variables.

MLRA was conducted to construct a regression model.

$\rho=\frac{1}{1+e^{-x}}; X=\beta_{0}+\sum_{i=0}^{np}\beta_{i}x_{i},$

where X presents a linear combination of selected variables (χ_i_) (i=1~np), and β_i_ is a partial regression coefficient for the i-th variable predicted by the maximum likelihood procedure. “$\rho$” represents the probability of belonging to the fatal group, calculated by assigning a set of the last IP test as an object variable. MLRA was used to estimate the intercept, regression coefficient, chi-square value, p-value, VIF, adjusted OR, 95% CI, AUC, AIC, sensitivity, and specificity.

For internal validation, bootstrap was employed by resampling with replacement from the derivation dataset 1,000 times, constructing ROC curves, calculating AUCs, and determining the mean and 95% CI of AUC values.

In addition, machine learning prediction models were established by inputting all raw data of the derivation dataset using Prediction One™ (Model 2) and LightGBM (Model 3).

Prediction One™ performed two-fold internal cross-validation and used “Neural Network Libraries” (https://nnabla.org) to adjust the weights and biases of variables. The degree of contribution of variables and contributive ranges to 72-hour outcomes were evaluated using a method of permutation feature importance. This involved calculating the model output difference when removing a single variable, indicating the model dependency. However, this method does not provide SHapley Additive exPlanation (SHAP) values.

The performance and feature importance of Model 3 were evaluated using SHAP values. Specifically, beeswarm summary plots were generated to visualize the impact of each feature on the model’s predictions, while a bar plot of mean SHAP values was used for feature ranking based on their average contribution. Additionally, SHAP dependence plots were employed to assess the relationship between individual features and the predicted outcome.

For internal validation, bootstrap resampling (n=1,000) was performed, following recommended best practices to enhance transparency in machine learning-based predictions using bootstrap simulation and SHAP analysis [31]. To comprehensively evaluate model performance, all three models and bootstrap resampling estimates were assessed using AUC, 95% CI of AUC, sensitivity, specificity, accuracy, precision, recall, F1 score, Log loss, Matthews correlation coefficient (MCC), and Cohen’s kappa.

External Validation

External validities were evaluated by applying each prediction model to the validation dataset and comparing their respective AUCs, 95% CI of AUCs, sensitivity, specificity, accuracy, precision, recall, F1 score, Log loss, MCC, and Cohen’s kappa.

Calibration Plot

Calibration plots were used to compare the predicted and actual probabilities of derivation and validation datasets.

Statistical Software

Data were analyzed using StatFlex software version 7.0.10 (Artech Co. Ltd., Osaka, Japan) and Prediction One™ for Models 1 and 2, respectively. MLRA bootstrap, LightGBM analysis, and calibration plots were performed on Google Colaboratory using the Python codes provided in the Supplemental File. G*Power version 3.1.9.4 [24] was used for sample size calculation. Python scripts used for the analyses are included in the Appendices.

## Results

Participant demographics

**Figure 2:**
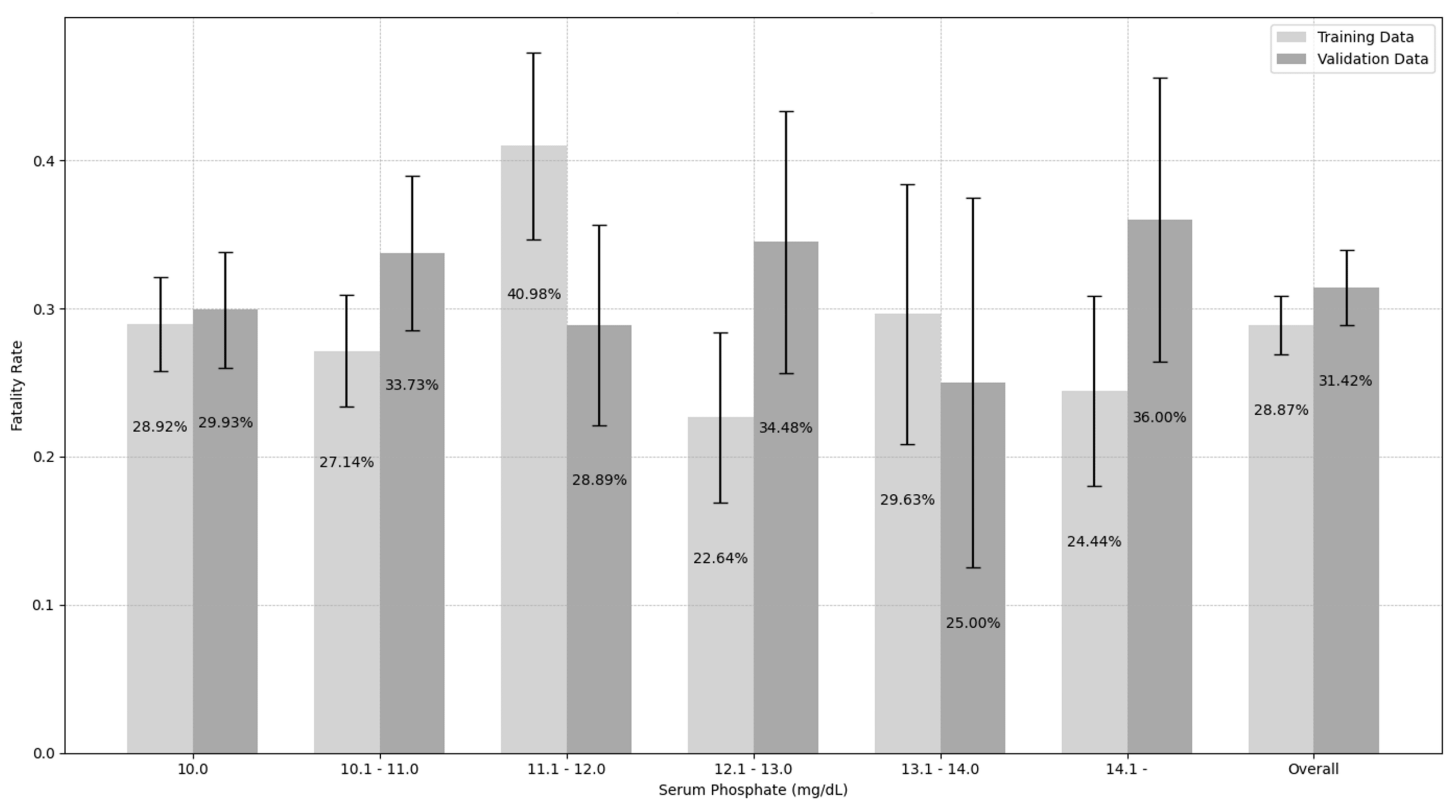
72-hour fatality rates stratified by serum phosphate levels in severe hyperphosphatemia patients. Fatality rates remain consistently high across all phosphate levels, with no clear dose-response relationship observed. Bars represent fatality rates for Dataset-A (light gray) and Dataset-B (dark gray) across different serum phosphate ranges. Overall fatality rates were 28.87% (153/530) for Dataset-A and 31.42% (104/331) for Dataset-B, demonstrating the severe prognosis associated with hyperphosphatemia ≥10 mg/dL.

The study group characteristics are presented in Table 1. No significant difference was observed in median IP levels between fatalities and survivors. The mortality rates classified by phosphorus concentration were similar across all concentrations (Figure 2). The overall fatality rates were identical, at 28.9% (153/530) and 31.4% (104/331) for training and validation data, respectively.

**Table 1:** Characteristics, patient demographics, vital signs, laboratory test values, and updated CCI of the derivation (Dataset-A). Continuous variables were expressed as medians and interquartile ranges. *P*-values were calculated using Fisher’s exact test for categorical variables and the Mann-Whitney U-test for continuous variables. APTT, activated partial thromboplastin time; CCI, Charlson comorbidity index; FDP, fibrin/fibrinogen degradation products; HDL-C, high-density lipoprotein cholesterol; N/A, not applicable; PT-INR, prothrombin time-international normalized ratio; IP, inorganic phosphorus

Variables	Unit	Total	Death (n=153)	N	Survival (n=377)	N	P-value
Age	years	530	70.0 (59.0-81.0)	153	64.0 (52.0-74.0)	377	0.000
Body mass index	kg/m^2^	491	21.6 (18.5-25.3)	129	21.6 (19.2-24.4)	362	0.711
Brinkman index	cigarettes/day×years	453	205 (0-630)	116	400 (0-800)	337	0.024
Males	%	530	66.0	101	72.9	275	0.111
Total updated CCI	points	530	5.0 (3.0-7.0)	153	3.0 (1.0-4.0)	377	0.000
Vital signs							
Body temperature	°C	455	36.3 (35.3-37.0)	142	36.5 (36.0-37.0)	313	0.065
Diastolic blood pressure	mmHg	514	55.0 (40.0-67.3)	145	70.0 (56.0-84.0)	369	0.000
Pulse rate	beats/min	516	91.5 (76.0-110.0)	150	88.0 (75.0-100.0)	248	0.015
Respiratory rate	breath/min	393	22.0 (18.0-29.8)	135	20.0 (17.0-25.0)	258	0.004
Systolic blood pressure	mmHg	522	92.0 (78.0-115.3)	149	122.0 (100.0-148.0)	373	0.000
Complete blood count							
Hematocrit	%	530	28.70 (23.20-36.65)	153	31.00 (26.10-37.10)	377	0.014
Hemoglobin	g/dL	530	9.30 (7.38-11.73)	153	10.10 (8.48-12.20)	377	0.003
Platelet count	10^4^/μL	530	14.30 (5.88-23.83)	153	19.90 (12.90-28.23)	377	0.000
Red blood cells	10^4^/μL	530	291.0 (231.8-379.5)	153	323.0 (275.0-395.5)	377	0.002
White blood cells	10^3^/μL	530	13.74 (7.84-20.11)	153	10.01 (6.85-15.04)	377	0.001
Biochemical examinations							
Alanine aminotransferase	U/L	513	92.0 (25.3-433.5)	151	23.0 (12.0-65.0)	362	0.000
Albumin	g/dL	500	2.60 (1.90-3.10)	138	3.20 (2.50-3.60)	362	0.000
Alkaline phosphatase	U/L	500	304.0 (214.3-478.5)	147	268.0 (183.0-376.0)	353	0.000
Amylase	U/L	265	143.0 (75.3-399.3)	83	138.5 (80.0-271.0)	182	0.487
Antithrombin Ⅲ	%	61	48.0 (33.3-72.8)	33	63.0 (42.0-90.5)	28	0.067
APTT	sec	390	55.20 (40.80-106.40)	134	38.75 (33.25-50.65)	256	0.000
Aspartate aminotransferase	U/L	513	250.0 (48.0-974.0)	151	26.0 (14.0-100.0)	362	0.000
Blood urea nitrogen	mg/dL	528	81.00 (47.00-140.00)	151	101.00 (71.75-136.00)	377	0.012
Calcium	mg/dL	530	7.50 (6.60-8.63)	153	7.80 (6.70-8.60)	377	0.337
Chloride	mEq/L	528	100.0 (96.0-105.0)	153	101.0 (96.0-106.0)	375	0.722
C-reactive protein	mg/dL	476	8.360 (2.458-14.538)	141	2.520 (0.423-8.540)	335	0.000
Creatinine	mg/dL	528	3.990 (2.303-7.053)	151	8.640 (4.295-12.723)	377	0.000
Creatine kinase	U/L	456	382.0 (171.5-1620.3)	139	225.0 (96.5-755.5)	317	0.000
D-dimer	μg/mL	239	12.40 (5.63-24.33)	87	6.10 (2.30-15.10)	152	0.000
Direct bilirubin	mg/dL	481	0.62 (0.21-1.79)	150	0.18 (0.10-0.46)	331	0.000
FDP	μg/mL	155	28.05 (11.30-55.60)	62	16.40 (6.08~34.78)	93	0.003
Fibrinogen	mg/dL	175	288.0 (192.8-410.8)	61	347.0 (246.0-546.0)	114	0.007
g-Glutamyl transferase	U/L	469	49.0 (22.0-136.0)	138	35.0 (19.0-82.5)	331	0.010
HDL-C	mg/dL	145	25.0 (16.5-37.8)	25	37.5 (27.5-51.0)	120	0.001
IP	mg/dL	530	11.40 (10.50-12.70)	153	11.30 (10.50-12.93)	377	0.943
Lactate dehydrogenase	U/L	506	861.0 (409.5-1956.5)	151	355.0 (238.0-570.8)	355	0.000
Magnesium	mg/dL	401	2.90 (2.50-3.33)	101	2.60 (2.20-3.00)	300	0.000
Potassium	mEq/L	528	6.30 (5.38-7.30)	153	5.40 (4.70-6.30)	375	0.000
PT-INR	N/A	393	1.550 (1.180-2.278)	133	1.160 (1.040-1.435)	260	0.000
Random plasma glucose	mg/dL	337	131.5 (87.0-181.0)	94	127.0 (98.0-178.8)	243	0.266
Sodium	mEq/L	528	139.0 (132.8-142.0)	153	137.0 (133.0-140.0)	375	0.202
Total bilirubin	mg/dL	481	1.00 (0.44-2.37)	150	0.38 (0.22-0.91)	331	0.000
Total cholesterol	mg/dL	240	131.5 (101.0-166.5)	64	164.5 (126.0~203.5)	176	0.001
Total protein	g/dL	499	5.50 (4.60-6.60)	137	6.30 (5.60-6.90)	362	0.000
Triglyceride	mg/dL	226	97.0 (60.8-167.0)	57	127.0 (81.8-179.5)	169	0.082
Uric acid	mg/dL	446	10.80 (8.10-14.60)	114	10.00 (7.80-12.90)	332	0.031

Demographic analysis revealed significant differences between fatalities and survivors. Fatalities were significantly older and had shorter stature and lower Brinkman index compared to survivors. Moreover, fatalities exhibited higher pulse and respiratory rates but lower SBP and DBP than survivors.

The expected causes of severe hyperphosphatemia varied between groups. Sepsis, pneumonia, malignancy, acute kidney disease, and hypovolemic shock were more frequently observed in fatalities. In contrast, chronic kidney disease was more prevalent among survivors. Additional results are shown in Table 2. Characteristics of Dataset-B are presented in Table 3.

**Table 2:** Characteristics, patient demographics, CCI components, underlying causes, and dialysis therapy status. *P*-values were calculated using Fisher’s exact test for categorical variables. ^#^Intermittent hemodialysis, peritoneal dialysis, and continuous renal replacement therapy. CCI, Charlson comorbidity index; DKA, diabetic ketoacidosis; HIV, human immunodeficiency virus; N, number; N/A, not applicable

Variables	Death (n=153)		Survival (n=377)		P-value
	N	%	N	%	
Components of updated CCI (points)					
Any malignancy, including leukemia and lymphoma (2)	23	15.0	39	10.3	0.128
Acquired immunodeficiency syndrome/HIV infection (4)	0	0.0	0	0.0	N/A
Chronic pulmonary disease (1)	13	8.5	23	1.8	0.321
Congestive heart failure (2)	44	28.8	79	21.0	0.054
Dementia (2)	2	1.3	6	1.6	1.000
Diabetes with chronic complications or history of DKA (1)	18	11.8	102	27.1	0.000
Hemiplegia or paraplegia (2)	2	1.3	4	1.1	1.000
Metastatic solid tumor (6)	16	10.5	7	9.0	0.000
Mild liver disease (2)	28	18.3	44	11.7	0.044
Mild to severe renal disease (1)	110	71.9	324	85.9	0.000
Moderate or severe liver disease (4)	78	51.0	66	17.5	0.000
Rheumatologic disease (1)	9	5.9	13	3.4	0.203
Expected causes of extreme hyperphosphatemia					
Acute kidney injury	87	56.9	125	33.2	0.000
Any malignancy	36	23.5	33	8.8	0.000
Cerebrovascular diseases	19	12.4	43	11.4	0.742
Chronic kidney disease	64	41.8	245	65.0	0.000
Diabetic ketoacidosis	1	0.7	13	3.4	0.077
Gastrointestinal bleeding	4	2.6	7	9.0	0.523
Gastrointestinal perforation or obstruction	4	2.6	13	3.4	0.788
Heart failure	9	5.9	41	10.9	0.075
Hypovolemic shock	13	8.5	5	1.3	0.000
Hypothermia	8	5.2	1	3.4	0.341
Pneumonia	14	9.2	14	3.7	0.011
Sepsis	31	20.3	34	9.0	0.000
Other infectious diseases	5	3.3	13	3.4	0.917
Others	12	7.8	50	9.0	0.079
Dialysis therapy^#^ status					
Pre-implemented before extreme hyperphosphatemia	26	17.0	134	35.5	0.000
Post-initiated after extreme hyperphosphatemia	48	31.4	249	66.0	0.000

**Table 3:** Characteristics, patient demographics, and laboratory test values of the validation (Dataset-B). Continuous variables were expressed as medians and interquartile ranges. P-values were calculated using Fisher’s exact test for categorical variables and the Mann-Whitney U-test for continuous variables. ^#^intermittent hemodialysis, peritoneal dialysis, and continuous renal replacement therapy. APTT, activated partial thromboplastin time; FDP, fibrinogen-fibrin degradation product; HDL-C, high-density lipoprotein cholesterol; N, number; N/A, not applicable; PT-INR, prothrombin time-international normalized ratio; IP, Inorganic phosphorus

Variables	Unit	Total	Death (n=104)	N	Survival (n=227)	N	P-value
Age	years	331	75.0 (61.0-82.5)	104	67.0 (53.3-78.0)	227	0.001
Males	%	235	69.2	72	71.8	163	0.562
Complete blood count							
Hematocrit	%	330	27.50 (22.20-34.9)	103	31.60 (26.63-38.38)	227	0.000
Hemoglobin	g/dL	330	8.70 (6.90-11.28)	103	10.10 (8.50-12.10)	227	0.000
Platelet count	10^4^/μL	330	10.60 (4.35-17.18)	103	19.40 (13.73-29.18)	227	0.000
Red blood cells	10^4^/μL	330	282.0 (220.0-363.8)	103	331.0 (280.0-406.8)	227	0.000
White blood cells	10^3^/μL	330	11.44 (7.04-17.80)	103	13.36 (6.79-16.04)	227	0.431
Biochemical examinations							
Alanine aminotransferase	U/L	319	105.5 (25.0-504.0)	102	21.0 (12.0-48.0)	217	0.000
Albumin	g/dL	312	2.20 (1.80-2.78)	95	3.10 (2.30-3.50)	217	0.000
Alkaline phosphatase	U/L	324	297.5 (208.0-526.0)	104	268.0 (204.0-361.0)	220	0.033
Amylase	U/L	100	147.0 (57.0-305.5)	32	138.5 (87.5-240.5)	68	0.994
Antithrombin Ⅲ	%	12	49.0 (32.8-62.8)	7	98.0 (57.8-110.3)	5	0.048
APTT	sec	268	64.80 (38.90-132.90)	94	37.00 (30.40-50.60)	174	0.000
Aspartate aminotransferase	U/L	319	240.5 (44.0-1509.0)	102	29.0 (15.0-75.0)	217	0.000
Blood urea nitrogen	mg/dL	330	92.5 (47.5-133.0)	104	106.0 (71.00-134.00)	226	0.037
Calcium	mg/dL	331	7.50 (6.95-8.50)	104	8.00 (7.20-8.60)	227	0.048
Chloride	mEq/L	331	101.0 (95.0-107.0)	104	101.0 (96.0-105.0)	227	0.138
C-reactive protein	mg/dL	309	5.73 (2.46-13.28)	98	3.26 (0.97-9.17)	211	0.000
Creatinine	mg/dL	330	4.155 (2.375-7.625)	104	8.555 (3.660-12.450)	226	0.000
Creatine kinase	U/L	288	66.0 (118.5-2,226.5)	92	248.0 (107.0-691.0)	196	0.010
D-dimer	μg/mL	125	15.45 (6.30-44.50)	44	6.70 (2.18-15.08)	81	0.001
Direct bilirubin	mg/dL	308	0.670 (0.200-1.760)	102	0.190 (0.100-0.400)	206	0.000
FDP	μg/mL	43	45.25 (17.25-143.45)	24	19.70 (10.40-37.18)	19	0.061
Fibrinogen	mg/dL	145	271.1 (128.3-417.5)	59	98.0 (57.8-110.3)	86	0.000
g-Glitamyl transferase	U/L	309	52.5 (25.0-504.0)	100	33.0 (20.0-98.0)	209	0.039
HDL-C	mg/dL	67	146.0 (104.0-173.0)	10	36.0 (29.0-45.5)	57	0.181
IP	mg/dL	331	11.35 (10.60-12.55)	104	11.20 (10.43-12.58)	227	0.467
Lactate dehydrogenase	U/L	316	898.0 (411.0-2,705.0)	102	347.0 (240.0-513.0)	214	0.000
Magnesium	mg/dL	296	2.80 (2.40-3.28)	91	2.70 (2.20-3.10)	205	0.032
Potassium	mEq/L	331	6.30 (5.55-7.40)	104	5.50 (4.70-6.30)	227	0.000
PT-INR	N/A	229	1.775 (1.235-2.970)	84	1.160 (1.030-1.488)	145	0.000
Random plasma glucose	mg/dL	180	108.0 (71.5.0-176.8)	41	137.0 (94.3-211.8)	139	0.090
Sodium	mEq/L	331	140.5 (136.0-146.0)	104	138.0 (134.0-142.0)	227	0.002
Total bilirubin	mg/dL	308	0.985 (0.370-2.570)	102	0.400 (0.220-0.710)	206	0.000
Total cholesterol	mg/dL	95	151.0 (108.8-189.3)	19	151.5 (127.0-184.5)	76	0.665
Total protein	g/dL	312	4.90 (4.13-6.10)	95	6.30 (5.60-6.93)	217	0.000
Triglyceride	mg/dL	94	96.0 (41.3-223.8)	17	113.0 (84.8-187.0)	77	0.171
Uric acid	mg/dL	238	11.00 (8.63-15.28)	63	10.40 (7.83-14.10)	175	0.387
Dialysis therapy^#^ status			Death (n=104)		Survival (n=227)		P-value
			N	%	N	%	
Pre-implemented before extreme hyperphosphatemia			21	20.2	32	30.8	0.773
Post-initiated after extreme hyperphosphatemia			49	21.6	137	60.4	0.000

Table 4 presents the proportion of patients who underwent dialysis before and after the onset of severe hyperphosphatemia in Dataset-A and Dataset-B. The proportion of patients who received dialysis before the onset of severe hyperphosphatemia differed significantly between Dataset-A and Dataset-B (p=0.001), whereas no significant difference was observed between deceased and surviving patients within each dataset (p=0.487). Similarly, the proportion of patients who initiated dialysis after the onset of severe hyperphosphatemia differed significantly between Dataset-A and Dataset-B (p=0.009), but no significant difference was found between deceased and surviving patients (p=0.982). These findings indicate that dialysis initiation patterns varied between datasets, whereas pre- and post-implemented dialysis did not significantly impact survival outcomes within each dataset.

**Table 4:** Proportion of patients receiving dialysis indicating intermittent hemodialysis, peritoneal dialysis, and continuous renal replacement therapy initiated before and after severe hyperphosphatemia between Dataset-A and Dataset-B. ^#^intermittent hemodialysis, peritoneal dialysis, and continuous renal replacement therapy *Chi-square test n, number

Dialysis therapy^#^ status	Death (n/total death)	%	Survival (n/total survival)	%	P-value* between Dataset-A and Dataset-B
Pre-implemented before extreme hyperphosphatemia					
Dataset-A	26/153	17.0	134/377	35.5	0.001
Dataset-B	21/104	20.2	32/227	30.8	
P-value* between death and survival	0.487				
Post-initiated after extreme hyperphosphatemia					
Dataset-A	48/153	31.4	249/377	66.0	0.009
Dataset-B	49/104	21.6	137/227	60.4	
P-value* between death and survival	0.982				

Univariate and multivariate logistic regression analyses

The crude OR and OR adjusted for age and IP levels revealed that age, PR, RR, WBC, T-Bili, D-Bili, AST, ALT, γ-GTP, LD, ALP, CK, CRP, K, Mg, PT-INR, APTT, FDP, D-dimer, and CCI total score significantly increased the risk of 72-hour fatality. Conversely, the Brinkman index, SBP, DBP, RBC, Hb, Ht, Plt, TP, ALB, BUN, Cr, TC, HDL-C, RPG, and fibrinogen demonstrated protective effects (Table 5).

**Table 5:** Univariate logistic regression analysis and MLRA results. To calculate the OR, the variations of sodium and chloride were 0.1, whereas the variations of others were 1.0. AIC, Akaike's information criterion; ALP, alkaline phosphatase; ALT, alanine transaminase; APTT, activated partial thromboplastin time; AST, aspartate transaminase; AUC, area under the curve; BUN, blood urea nitrogen; CCI, Charlson comorbidity index; CK, creatine kinase; CRP, C-reactive protein; DBP, diastolic blood pressure; D-Bili, direct bilirubin; FDP, fibrin/fibrinogen degradation products; g-GTP, g-glutamyl transpeptidase; Hb, hemoglobin; HDL-C, high-density lipoprotein cholesterol; Ht, hematocrit; LD, lactate dehydrogenase; OR, odds ratio; PT-INR, prothrombin time-international normalized ratio; RBC, red blood cell; RPG, random plasma glucose; RR, respiratory rate; SBP, systolic blood pressure; TC, total cholesterol; TG, triglyceride; TP, total protein; T-Bili, total bilirubin; WBC, white blood cell; MLRA, multivariate logistic regression analysis; IP, inorganic phosphorus

Variables	Univariate logistic regression analysis			MLRA				
	Crude OR (95% CI)	P-value	AUC	Adjusted OR (95% CI) by age and IP	P-value	AUC	AIC	n (death: survival)
Age (years)	1.028 (1.014-1.041)	0.0001	0.620	1.028 (1.014-1.042)	0.0001	0.620	626.01	530 (153:377)
Brinkman index	1.000 (0.999-1.000)	0.0493	0.568	1.000 (0.999-1.000)	0.0186	0.618	507.12	453 (116:337)
Total updated CCI	1.478 (1.347-1.622)	0.0000	0.745	1.467 (1.336-1.612)	0.0000	0.764	552.31	530 (153:377)
Vital signs								
DBP (mmHg)	0.967 (0.956-0.977)	0.0000	0.695	0.968 (0.958-0.979)	0.0000	0.706	563.15	514 (145:369)
PR (beats/mins)	1.011 (1.002-1.019)	0.0119	0.568	1.015 (1.006-1.024)	0.0011	0.648	601.45	516 (150:366)
RR (breath/min)	1.039 (1.009-1.070)	0.0099	0.700	1.041 (1.011-1.072)	0.0077	0.629	498.80	393 (135:258)
SBP (mmHg)	0.973 (0.966-0.979)	0.0000	0.737	0.972 (0.964-0.979)	0.0000	0.757	540.53	522 (149:373)
Complete blood count								
Ht (%)	0.973 (0.952-0.994)	0.0107	0.568	0.968 (0.947-0.990)	0.0041	0.639	619.34	530 (153:377)
log (Hb (g/dL))	0.357 (0.193-0.661)	0.0010	0.568	0.330 (0.175-0.622)	0.0006	0.644	615.79	530 (153:377)
Platelet (10^4^/μL)	0.963 (0.947-0.980)	0.0000	0.639	0.962 (0.946-0.979)	0.0000	0.665	606.82	530 (153:377)
RBC (10^4^/μL)	0.997 (0.995-0.999)	0.0070	0.588	0.997 (0.995-0.999)	0.0045	0.646	619.51	530 (153:377)
WBC^0.5^ (10^3^/μL)	1.149 (1.056-1.250)	0.0013	0.588	1.152 (1.056-1.257)	0.0014	0.665	617.64	530 (153:377)
Biochemical examinations								
Albumin (g/dL)	0.394 (0.301-0.515)	0.0000	0.702	0.385 (0.290-0.510)	0.0000	0.738	523.67	500 (138:362)
BUN (mg/dL)	0.996 (0.992-1.000)	0.0409	0.570	0.995 (0.992-0.999)	0.0170	0.632	617.09	528 (151:377)
Calcium (mg/dL)	0.937 (0.823-1.067)	0.3258	0.527	0.907 (0.793-1.037)	0.1543	0.619	625.96	530 (153:377)
Chloride (mEq/L)	1.003 (0.982-1.024)	0.8096	0.510	0.999 (0.977-1.020)	0.8988	0.619	626.74	528 (153:375)
Creatinine (mg/dL)	0.827 (0.786-0.870)	0.0000	0.721	0.830 (0.788-0.874)	0.0000	0.731	557.02	528 (151:377)
log (ALP (U/L))	2.041 (1.450-2.873)	0.0000	0.605	2.100 (1.479-2.981)	0.0000	0.673	577.67	500 (147:353)
log (ALT (U/L))	1.621 (1.428-1.841)	0.0000	0.720	1.634 (1.435-1.860)	0.0000	0.749	551.64	513 (151:362)
log (APTT (second))	4.164 (2.730-6.350)	0.0000	0.719	4.402 (2.902-6.983)	0.0000	0.743	447.73	390 (134:256)
log (AST (U/L))	1.718 (1.525-1.936)	0.0000	0.776	1.746 (1.545-1.974)	0.0000	0.796	517.10	513 (151:362)
log (CK (U/L))	1.247 (1.119-1.391)	0.0000	0.611	1.316 (1.171-1.479)	0.0000	0.669	532.08	456 (139:317)
log (CRP (mg/dL))	1.429 (1.253-1.631)	0.0000	0.660	1.358 (1.211-1.585)	0.0000	0.686	543.77	476 (141:335)
log (D-Bili (mg/dL))	1.900 (1.614-2.237)	0.0000	0.729	2.010 (1.692-2.387)	0.0000	0.762	513.80	581 (150:331)
log (D-dimer (μg/mL))	1.499 (1.197-1.877)	0.0004	0.657	1.488 (1.187-1.865)	0.0006	0.656	305.77	239 (87:152)
log (FDP (μg/mL))	1.529 (1.143-2.045)	0.0043	0.642	1.525 (1.139-2.043)	0.0047	0.640	307.69	155 (62:93)
log (fibrinogen)	0.463 (0.280-0.766)	0.0027	0.623	0.449 (0.269-0.751)	0.0023	0.625	224.11	175 (61:114)
log (g-GTP (U/L))	1.315 (1.094-1.581)	0.0036	0.575	1.398 (1.154-1.693)	0.0006	0.646	548.85	469 (138:331)
log (HDL-C (mg/dL))	0.227 (0.095-0.543)	0.0009	0.705	0.184 (0.071-0.478)	0.0005	0.832	109.32	145 (25:120)
log (IP (mg/dL))	0.871 (0.413-1.836)	0.7171	0.502	0.993 (0.284-3.466)	0.9911	0.919	626.01	530 (153:377)
log (LD (U/L))	2.280 (1.857-2.799)	0.0000	0.745	2.531 (2.040-3.141)	0.0000	0.791	521.05	506 (151:355)
log (magnesium (mg/dL))	3.077 (1.745-5.428)	0.0001	0.624	3.020 (1.667-5.469)	0.0003	0.650	438.14	401 (101:300)
log (PT-INR)	2.052 (1.571-2.680)	0.0000	0.692	2.109 (1.604-2.771)	0.0000	0.705	470.38	393 (133:260)
log (RPG (mg/dL))	0.631 (0.457-0.872)	0.0052	0.539	0.656 (0.474-0.909)	0.0114	0.669	386.01	337 (94:243)
log (T-Bili (mg/dL))	1.961 (1.628-2.361)	0.0000	0.704	2.094 (1.722-2.547)	0.0000	0.743	525.58	481 (150:331)
log (T-CHO (mg/dL))	0.240 (0.107-0.540)	0.0006	0.644	0.260 (0.113-0.601)	0.0016	0.713	262.32	240 (64:176)
log (TG (mg/dL))	0.727 (0.477-1.109)	0.1388	0.567	0.895 (0.567-1.414)	0.6343	0.680	248.43	226 (57:169)
log (TP (g/dL))	0.073 (0.029-0.187)	0.0000	0.654	0.053 (0.020-0.143)	0.0000	0.713	536.40	499 (137:362)
log (uric acid (mg/dL))	1.504 (0.911-2.482)	0.1106	0.568	1.587 (0.938-2.868)	0.0853	0.645	494.64	446 (114:332)
Potassium (mEq/L)	1.557 (1.342-1.807)	0.0000	0.674	1.544 (1.321-1.806)	0.0000	0.688	594.84	528 (153:375)
Sodium (mEq/L)	1.021 (0.997-1.045)	0.0814	0.535	1.019 (0.995-1.043)	0.1238	0.620	624.38	528 (153:375)

Model 1

**Figure 3:**
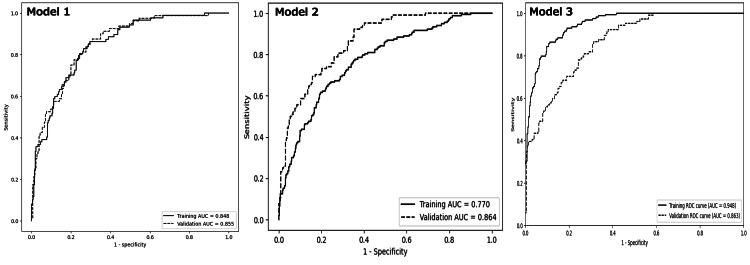
Comparison of receiver operating characteristic curves of three predicting models for the 72-hour fatality of patients with severe hyperphosphatemia ≥10 mg/dL between Dataset-A and Dataset-B. The receiver operating characteristic curves for Models 1, 2, and 3 were generated using Dataset-A for derivation and Dataset-B for validation. Black solid lines represent the prediction probabilities for the 72-hour fatality derived from Models 1, 2, and 3 trained on Dataset-A. Black dotted lines indicate the prediction probability for the 72-hour fatality validated using Dataset-B. AUC, area under the curve; ROC, receiver-operating characteristic

Five covariates, that is, age, ALB, AST, K, and Mg, were identified as the most significant predictors from all hematological and biochemical variables using backward elimination (Table 6). The C-statistics for this optimal combination of covariates in predicting 72-hour fatality was 0.848 (95% CI, 0.801-0.890) (Figure 3). The final predictive model for 72-hour fatality probability “p” calculated by inputting patient-specific variables is as follows:

$p=1/\left[ 1+exp\left( -5.836 + 0.031 \left( Age \right)- 1.050\left( ALB \right)+ 0.482log \left( AST \right)+ 0.296\left( K \right)+ 1.530log\left( Mg \right) \right) \right]$

**Table 6:** MLRA (Model 1) for predicting 72-hour fatality. AIC, Akaike information criterion; AST, aspartate transaminase; AUC, area under the curve; exp. var, explanatory variable; MLRA, multivariate logistic regression analysis; N/A, not applicable; obj. var, object variable; OR, odds ratio; SE, standard error; Sn, sensitivity; Sp, specificity; VIF, variance inflation factor; β: partial regression coefficient

MLRA: Obj. var=death, n=357 (with all 5 exp. vars)						
Exp. var	β	SE (β)	z	P	VIF	OR (95% CI)
Intercept	−5.836	1.151	N/A	N/A	N/A	N/A
Age (years)	0.031	0.010	3.008	0.003	1.059	1.032 (1.011-1.053)
Albumin (g/dL)	−1.050	0.210	−5.005	0.000	1.066	0.350 (0.232-0.0248)
log (AST (U/L))	0.482	0.083	5.779	0.000	1.091	1.618 (1.375-1.906)
Potassium (mEq/L)	0.296	0.114	2.600	0.009	1.082	1.344 (1.076-1.680)
log (magnesium (mg/dL))	1.530	0.562	2.721	0.007	1.062	4.616 (1.534-13.890)
AIC=295.821, AUC=0.848 (95% CI=0.801-0.890), Sn=0.862, Sp=0.704						

This model demonstrated a sensitivity of 86.2% and specificity of 70.4% in predicting 72-hour fatality for patients with extremely high IP levels.

Based on 1,000 bootstrap resampling iterations, the validation results indicated an average AUC of 0.848 (95% CI, 0.800-0.894), demonstrating good discriminatory performance. The model showed a sensitivity of 0.818 (95% CI, 0.686-0.908), a specificity of 0.743 (95% CI, 0.665-0.855), and an overall accuracy of 0.761 (95% CI, 0.706-0.824). Apparent performance closely matched corrected performance, with minimal optimism (0.001).

Model 2

The AUC of Model 2 was 0.803 (95% CI, 0.762-0.844) (Figure 3), with a recall of 0.706 and an F-score of 0.637. Prediction One™ calculated the degree of contribution of variables and the ranges of contributions to 72-hour outcomes. Results in descending order are shown in Table 7.

**Table 7:** Variables ranked by total degree of contribution and their most contributive ranges to 72-hour outcomes of Prediction One™. APTT, activated partial thromboplastin time; PT-INR, prothrombin time-international normalized ratio; FDP, fibrin/fibrinogen degradation products; HDL-C, high-density lipoprotein cholesterol

No	Variables	Total degree of contribution	Most contributing ranges for 72-hour fatalities	Most contributing ranges for 72-hour survival
1	AT-3 (%)	0.068583	55.0-67.0	64.0-2.0
2	Hematocrit (g/dL)	0.062246	7.6-20.2	39.7-45.6
3	APTT (seconds)	0.061941	200.0-200.0	33.7-36.8
4	Albumin (g/dL)	0.058494	N/A	2.7-3.1
5	C-reactive protein (mg/dL)	0.058233	9.40-13.95	0.46-1.09
6	Creatinine (mg/dL)	0.057596	3.91-5.33	8.67-10.41
7	Magnesium (mg/dL)	0.056854	N/A	2.6-2.8
8	Total protein (g/dL)	0.055029	N/A	6.7-7.0
9	Age (years)	0.053129	88.0-93.0	57.0-62.0
10	Potassium (mEq/L)	0.052290	7.10-8.00	2.90-4.20
11	PT-INR	0.052288	1.62-2.19	1.26-1.42
12	Blood urea nitrogen (mg/dL)	0.051575	175.0-301.0	131.0-152.0
13	Alkaline phosphatase (U/L)	0.050601	243.1-285.1	285.1-327.1
14	Phosphorus (mg/dL)	0.049768	12.1-13.2	14.7-26.9
15	Aspartate transaminase (U/L)	0.049576	163.1-466.5	25.1-38.9
16	D-dimer (μg/mL)	0.049427	8.7-13.0	2.8-4.4
17	Alanine transaminase (U/L)	0.049401	112.3-242.1	34.0-57.5
18	Uric acid (mg/dL)	0.049291	N/A	8.4-9.3
19	Amylase (U/L)	0.048598	653.3-5925.0	269.5-410.2
20	Lactate dehydrogenase (U/L)	0.046622	2,709.3-37,560.0	290.0-349.9
21	FDP (μg/mL)	0.045347	11.0-14.5	1.0-5.0
22	Platelet (/μL)	0.043840	3,000-50,062	127,000-155,022
23	Direct bilirubin (mg/dL)	0.043217	11.24-34.15	0.29-0.53
24	Hemoglobin (g/dL)	0.043205	8.6-9.4	11.0-12.0
25	Chloride (mEq/L)	0.042890	97.0-99.0	104.0-107.0
26	Sex	0.042745	0 (Female)	1 (Male)
27	Fibrinogen (mg/dL)	0.041850	207.1-258.1	784.1-1,562.0
28	White blood cells (/μL)	0.041709	25,940-67,390	15,462-18,723
29	Total bilirubin (mg/dL)	0.041390	1.76-3.95	0.30-0.39
30	HDL-C (mg/dL)	0.041033	5.0-18.0	45.0-51.0
31	Sodium (mEq/L)	0.040714	150.0-174.0	136.0-138.0
32	Creatine kinase (U/L)	0.038621	9,785.5-210,300.0	847.1-1,625.2
33	Red blood cell (×10^4^/μL)	0.038182	41.0-272.1	321.1-345.0
34	γ-Glutamyl transferase (U/L)	0.035741	270.1-2,071.0	6.0-13.0
35	Random Plasma Glucose (mg/dL)	0.034261	185.0-244.1	136.1-152.1

Model 3

**Figure 4:**
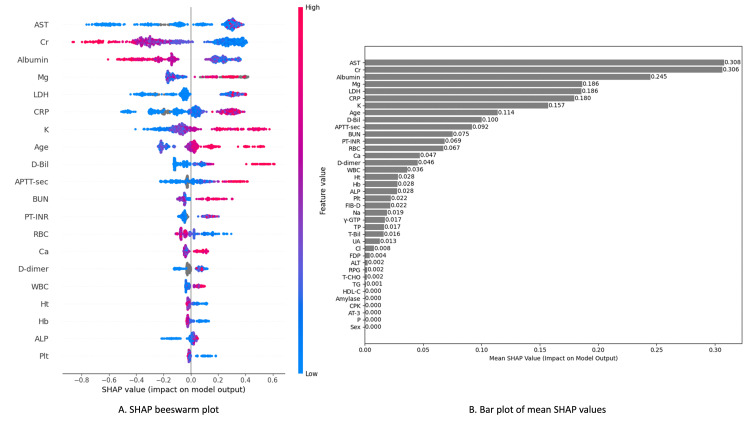
SHAP beeswarm summary plots and bar plots of mean SHAP values for Model 3. SHAP beeswarm plot shows a dense summary of how the top features in the derivation dataset impact predictions for 72-hour fatality. Each feature row corresponds to a specific feature, with each dot representing an instance. The SHAP value of that feature determined the × position of the dot, and dots pile up along each feature row to show density (A). The “bar plot of mean SHAP values” shows the average SHAP values for each explanatory variable in descending order, offering an overview of feature importance (B). ALP, alkaline phosphatase; ALT, alanine transaminase; APTT, activated partial thromboplastin time; AST, aspartate transaminase; BUN, blood urea nitrogen; Cr, creatinine; CRP, C-reactive protein; D-Bili, direct bilirubin; FDP, fibrinogen degradation products; GTP, glutamyl transpeptidase; Hb, hemoglobin; HDL-C, high-density lipoprotein cholesterol; Ht, hematocrit; LDH, lactate dehydrogenase; Plt, platelet count; PT-INR, prothrombin time-international normalized ratio; RBC, red blood cell; RPG, random plasma glucose; SHAP, SHapley Additive exPlanation; T-Bili, total bilirubin; UC, uric acid; WBC, white blood cell

**Figure 5:**
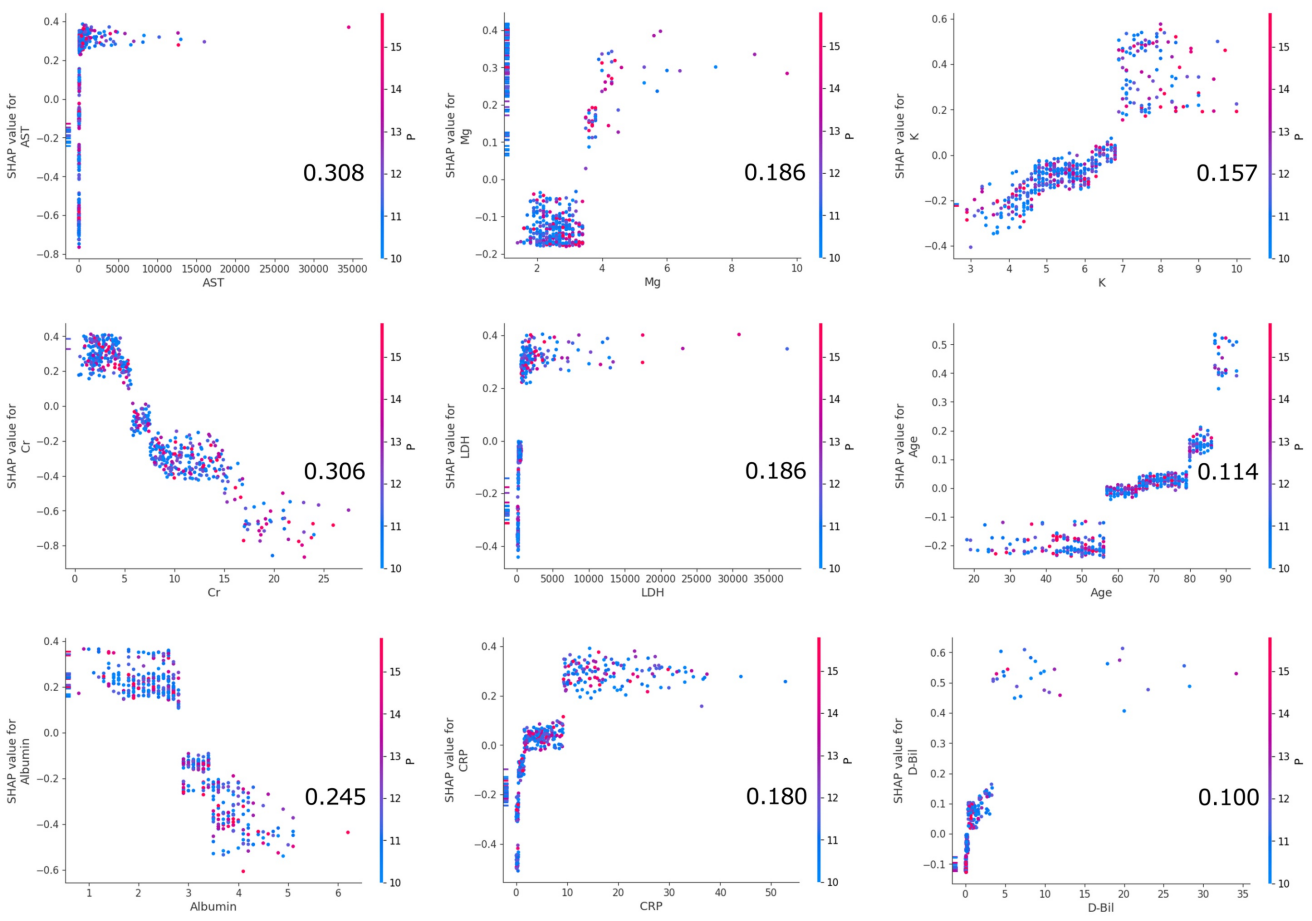
SHAP dependence plots for the top nine features. The figure shows SHAP dependence plots for the nine most influential variables in predicting the 72-hour mortality rate based on LightGBM analysis. AST, Cr, and ALB have the highest SHAP values, indicating they contribute the most to the model’s prediction. Higher levels of AST and Cr are associated with increased mortality risk, while lower levels of albumin are linked to a higher risk. Mg, LDH, and CRP also show significant impacts, with higher values leading to increased mortality risk. K, age, and D-Bili have a moderate influence, where higher levels are associated with a greater likelihood of mortality. The color gradient in each plot represents the values of these features, with red indicating higher feature values and blue representing lower ones. AST, aspartate transaminase; ALB, albumin; Cr, creatinine; CRP, CRP, C-reactive protein; D-Bili, direct bilirubin; LDH, lactate dehydrogenase; SHAP, SHapley Additive exPlanation; D-Bili, direct bilirubin

Model 3 attained a C-statistic of 0.948 (95% CI, 0.923-0.973) (Figure 3). The analysis using the SHAP method identified baseline AST, Cr, ALB, Mg, LD, CRP, K, age, and D-Bili as the top nine influential variables in predicting 72-hour fatality (Figure 4 and Figure 5).

External validation

An independent dataset (Dataset-B; n=331; 104 deceased, 227 alive) was used to validate Models 1, 2, and 3 externally and compute 72-hour fatality probability. The dataset achieved predictive accuracies of 0.855 (95% CI, 0.809-0.899), 0.864 (95% CI, 0.809-0.899), and 0.863 (95% CI, 0.816-0.911), respectively. All models exhibited robust discrimination performance for 72-hour fatality in external validation (Table 8).

**Table 8:** Comparison of statistical markers of three models. AUC, area under the curve; LightGBM, light gradient boosting machine; MCC, Matthews correlation coefficient; MLRA, multivariate logistic regression analysis

Metrics	MLRA (Model 1)	Prediction One™ (Model 2)	LightGBM (Model 3)
Derivation (Dataset-A)			
AUC (95% CI)	0.848 (0.801-0.890)	0.770 (0.722-0.818)	0.948 (0.923-0.973)
Sensitivity	0.862	0.653	0.863
Specificity	0.704	0.769	0.889
Accuracy	0.739	0.736	0.874
Precision	0.481	0.535	0.884
Recall	0.851	0.654	0.647
F1 score	0.614	0.588	0.747
Log loss	0.400	0.514	0.324
MCC	0.480	0.401	0.600
Cohen’s kappa	0.440	0.397	0.666
Validation (Dataset-B)			
AUC (95% CI)	0.855 (0.809-0.899)	0.864 (0.816-0.911)	0.863 (0.816-0.911)
Sensitivity	0.875	0.923	0.865
Specificity	0.692	0.648	0.687
Accuracy	0.743	0.734	0.785
Precision	0.548	0.545	0.739
Recall	0.863	0.923	0.490
F1 score	0.700	0.686	0.590
Log loss	0.431	0.422	0.426
MCC	0.510	0.531	0.470
Cohen’s kappa	0.477	0.481	0.452

Calibration plot

**Figure 6:**
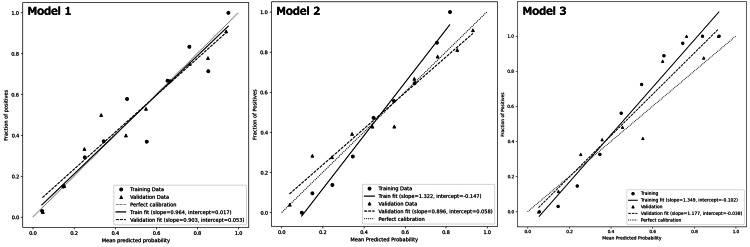
Comparison of calibration plots between the predicted probabilities of Models 1, 2, and 3 and the actual probability. The mean predicted probabilities (X-axis) for Models 1, 2, and 3 were assessed in a calibration plot to determine whether they matched the actual probability (Y-axis) of derivation and validation datasets. Black solid lines with black solid circles indicate the correlation between the predicted probabilities (X-axis) of Models 1, 2, and 3 and the actual probabilities (Y-axis) of the derivation dataset. Black dotted lines with black solid triangles indicate the correlation between the predicted probabilities (X-axis) of Models 1, 2, and 3 and the actual probability (Y-axis) of the validation dataset. The black dashed line indicates Y=X, representing perfect alignment between predicted and actual probabilities.

Regression lines for Models 1, 2, and 3 exhibited intercepts and slopes of 0.017 and 0.964, −0.147 and 1.322, and −0.102 and 1.349, respectively (Figure 6). The MLRA model demonstrated superior stability and reliability compared to others. Further analysis of external validation results revealed that the calibration plots for Models 1, 2, and 3 exhibited intercepts and slopes of 0.053 and 0.903, 0.058 and 0.896, and −0.038 and 1.177, respectively. All models demonstrated reasonable calibration in the external dataset. MLRA had the closest regression coefficient to 1.0 at 0.903.

## Discussion

Our investigation yielded four primary findings: 1) The overall 72-hour fatality rates were similar, at 28.9% (153/530) for training and 31.4% (104/331) for validation data. 2) Five statistically independent factors associated with 72-hour fatality were identified: advanced age, hypoalbuminemia, elevated AST, hyperkalemia, and hypermagnesemia. 3) Model 1 predicted 72-hour fatality with good discrimination and calibration. 4) Models 2 and 3 predicted 72-hour fatality with good discrimination and fair calibration but slightly overestimated the probabilities.

To our knowledge, this is the first study to report early fatality in patients with severe hyperphosphatemia. The observed 72-hour fatality rate of 28.9% underscored the gravity of this condition. This rate exceeded that reported for extreme hyperglycemia (≥500 mg/dL; 4.6%, 16/351) [3] and severe elevation of CRP (≥40 mg/dL; 16.0%, 44/275) [4]; however, it was lower than the fatality rate for severe AST elevation (≥3,000 U/L; 31.1%, 133/428) [5]. We found no significant difference in median phosphorus levels between fatality and survival groups, with mortality remaining constant across concentration levels. These findings highlight the complexity of predicting mortality based solely on severe hyperphosphatemia and underscore the need for more sophisticated predictive models to identify high-risk patients within this critical timeframe. Moreover, the five identified factors may serve as prognostic indicators and potential therapeutic targets, reinforcing their importance in risk stratification and clinical implications for patient management.

Numerous studies have reported age as an independent risk factor for adverse outcomes across several medical conditions [32], including hyperphosphatemia. One study reported a 28-day fatality rate of 10.7% in critically ill patients, including those with phosphate metabolism disorders [8]. This increasing vulnerability may be attributed to age-related physiological changes, including decreased organ reserve, impaired immune response, and diminished metabolic capacity.

Hypoalbuminemia has been widely associated with poor outcomes in several critical illnesses, including sepsis, liver disease, and renal failure requiring dialysis. Zitt et al. reported an inverse association between ALB levels and mortality in patients with incident dialysis, noting increased mortality risk with low or high phosphorus and ALB [33]. Hypoalbuminemia likely reflects systemic inflammation, compromising patients’ ability to fight severe hyperphosphatemia.

Elevated AST levels were independently associated with increased fatality in patients with severe hyperphosphatemia. High AST levels have low specificity for a single disease, as AST is found in cells of different organs [34]; however, it may reflect acute cellular damage. Along with ALP, CK, sodium, and potassium, IP was associated with 72-hour mortality in a study of cases with extremely high AST (≥3,000 U/L) [5], suggesting a bidirectional relationship between AST and IP levels in cellular damage.

Hyperkalemia and hyperphosphatemia are common complications of TLS [35]. Hyperkalemia from increased potassium intake, enhanced release from cells, or impaired urinary excretion [36] can lead to cardiac arrhythmias and impaired neuromuscular function. Research has linked hyperkalemia to worse outcomes among patients with extremely high serum AST (≥3,000 U/L) [5].

Hypermagnesemia was identified as another independent predictor of increased fatality in severe hyperphosphatemia. This finding contrasts with reports of reduced risk of cardiovascular mortality associated with hyperphosphatemia in patients with high serum magnesium [37]. Excessive magnesium can lead to cardiac conduction abnormalities, respiratory depression, and neuromuscular dysfunction [38]. The association between hypermagnesemia or hyperkalemia and poor outcomes emphasizes the importance of vigilant monitoring and appropriate management of electrolyte imbalances in these patients.

Statistical and machine learning approaches exhibited good discriminatory ability, as measured by the AUC, and can serve as valuable tools for fatality assessment. However, although machine learning is widely used in clinical practice, calibration, or evaluating the reliability of risk predictions, it is largely neglected. In addition, although class imbalance is frequently addressed, adjustments to correct it can distort prevalence and lead to inadequate risk predictions. Furthermore, machine learning methods require larger datasets [39] than MLRA. In our study, the dataset had a fatality rate of approximately 30% and a small class imbalance. Although the sample size was relatively modest (530 patients), we addressed potential overfitting at the model generation stage using cross-validation in Prediction One™ and by adjusting hyperparameters in LightGBM. Calibration for each model was acceptable, and validation results were good.

MLRA offers greater transparency and interpretability than machine learning for practical use, providing coefficients and OR for each variable that can be easily understood and compared. In contrast, machine learning is considered a “black box” that does not explicitly explain or justify its predictions, even when providing the descending order of variables with the best contributive variable ranges and SHAP values.

The current study has several limitations. First, our study findings may not be generalized to other populations or settings. Second, we selected patients with IP levels ≥10 mg/dL; factors associated with 72-hour fatality may differ in patients with elevated IP levels below this threshold. Third, unmeasured confounders and mediators of fatality, such as nutritional status and medication use, may have influenced the outcomes. Moreover, a pathophysiological veritable mechanism underlying the association between the 72-hour fatality and identical risk in patients with severe hyperphosphatemia remains unknown. However, the factors derived by MLRA can serve as a valuable reference. Furthermore, machine learning-based models suggest the possibility of creating predictive models at individual facilities. Future studies should explore the impact of these factors on outcomes and whether modifying them or administering hyperphosphatemia medications such as tenapanor can improve survival [40].

This study compared MLRA and machine learning approaches to predict 72-hour fatality in patients with severe hyperphosphatemia. Both methods were accurate; however, machine learning models showed slightly poor calibration performance. The study findings underscore the importance of considering old age, hypoalbuminemia, elevated AST, hyperkalemia, and hypermagnesemia during risk stratification and managing patients with severe hyperphosphatemia. Future studies should explore the mechanisms linking these factors to fatalities and develop targeted interventions to improve patient outcomes. Additionally, machine learning-based predictive models show potential and warrant further research.

## Conclusions

This study demonstrated that severe hyperphosphatemia is associated with a high 72-hour mortality rate (~30%), with advanced age, hypoalbuminemia, elevated AST, hyperkalemia, and hypermagnesemia identified as key predictors. Among predictive models, LightGBM achieved the highest AUC (0.948), while MLRA demonstrated superior calibration with a regression coefficient closest to 1.0 (0.903). These findings highlight critical differences between acute and chronic hyperphosphatemia, with AKI patients exhibiting significantly higher mortality than those with CKD, likely due to acute electrolyte disturbances. Although the proportion of patients with pre-existing dialysis and post-hyperphosphatemia dialysis initiation was higher in the survival group, the difference between survivors and non-survivors was not statistically significant.

Our findings underscore the prognostic utility of routinely collected laboratory data in risk stratification and clinical decision-making for hyperphosphatemia management. While hemodialysis remains the cornerstone of treatment, adjunctive strategies such as phosphate binders and dietary restrictions may offer additional benefits in chronic settings. This single-center study demonstrates that traditional statistical models remain clinically valuable for mortality prediction, though machine learning approaches show promise for refinement. Future multi-center research should validate these models across diverse populations and develop integrated management strategies addressing both acute and long-term complications of hyperphosphatemia.

## Appendices

Supplementary files

The provided materials include a detailed analysis protocol and code for conducting comparative analyses using multivariate logistic regression and machine learning methods (https://zenodo.org/records/15016255).
